# Frequent in dementia, deadliest without it: delirium and mortality in hospitalised older adults

**DOI:** 10.1093/ageing/afag081

**Published:** 2026-04-09

**Authors:** Mfon Umoh, Thiago J Avelino-Silva, Marlon Juliano Romero Aliberti, Flavia Barreto Garcez, Sei J Lee, Alexander K Smith, Esther Oh

**Affiliations:** Division of Geriatric Medicine and Gerontology, Department of Medicine, Johns Hopkins School of Medicine, Baltimore, MD 21224, USA; Division of Geriatrics, Department of Medicine, University of California San Francisco, San Francisco, CA 94143, USA; Laboratorio de Investigacao Medica em Envelhecimento (LIM-66), Servico de Geriatria, Hospital das Clinicas da Faculdade de Medicina da Universidade de Sao Paulo, São Paulo, SP, 05403-000, Brazil; Laboratorio de Investigacao Medica em Envelhecimento (LIM-66), Servico de Geriatria, Hospital das Clinicas da Faculdade de Medicina da Universidade de Sao Paulo, São Paulo, SP, 05403-000, Brazil; Research Institute, Hospital Sirio-Libanes, São Paulo, SP, 01308-060, Brazil; Division of Geriatrics, Department of Medicine, Universidade Federal de Sergipe Hospital Universitario, Aracaju, SE, 49060-025, Brazil; Division of Geriatrics, Department of Medicine, University of California San Francisco, San Francisco, CA 94143, USA; Division of Geriatrics, Department of Medicine, University of California San Francisco, San Francisco, CA 94143, USA; Division of Geriatric Medicine and Gerontology, Department of Medicine, Johns Hopkins School of Medicine, Baltimore, MD 21224, USA

**Keywords:** delirium, dementia, hospitalisation, mortality, prognosis, effect modification, older people

## Abstract

**Background:**

Delirium is common in hospitalised older adults and is associated with mortality. Whether this prognostic association varies by baseline cognition is uncertain. We evaluated the association between delirium and 90-day mortality and whether baseline cognitive status modified this relationship.

**Methods:**

We conducted a prospective, multicentre cohort study of adults aged ≥65 years admitted to 43 hospitals in five countries (Brazil, Angola, Chile, Colombia and Portugal; June 2022–December 2023). Delirium was assessed using the Confusion Assessment Method; cognitive status was measured using an informant-based Clinical Dementia Rating (CDR). Mortality within 90 days of admission was ascertained from hospital records, structured telephone follow-up by blinded assessors and registry linkage. We used mixed-effects survival models with random intercepts (state/province and study centre) and sequential adjustment for sociodemographic, clinical and hospital-related factors. Effect modification by CDR was examined with stratified analyses.

**Results:**

Among 2556 patients (mean age 79 ± 9 years; 56% women), delirium occurred in 957 (37%). Delirium frequency rose with worsening cognition (CDR 0: 16%; CDR 0.5: 27%; CDR 1: 59%; CDR 2–3: 77%; *P* < .001). Delirium was associated with higher 90-day mortality (adjusted HR = 3.45; 95% CI = 2.83–4.20). The relative association with mortality was greatest in no dementia and attenuated in moderate–severe dementia. At 90 days, cumulative mortality was 54% with delirium vs. 15% without in CDR 0 (HR = 4.40; 95% CI = 3.15–6.16) and 36% vs. 17% in CDR 2–3 (HR = 2.22; 95% CI = 1.34–3.66). Patients with delirium also experienced more in-hospital complications (nosocomial infection, functional decline and prolonged stay).

**Conclusions:**

Although delirium was more frequent among patients with dementia, its relative association with 90-day mortality was strongest in those with no baseline dementia. The results provide a strong rationale for intervention trials to determine whether delirium prevention and management strategies can reduce mortality, particularly among patients without known dementia.

## Key Points

- In 2556 adults ≥65 years across 43 hospitals, delirium occurred in 37% and predicted higher 90-day mortality (adjusted HR = 3.45; 95% CI = 2.83–4.20).
- The relative association between delirium and mortality was greatest with no dementia (CDR 0: HR = 4.40; 95% CI = 3.15–6.16) and attenuated with greater dementia severity (CDR 2–3: HR = 2.22; 95% CI = 1.34–3.66).
- Delirium is a high-risk prognostic marker across the cognitive spectrum, demonstrating the need for intervention trials to determine whether delirium prevention and management strategies reduce mortality, particularly among patients without known dementia.

## Introduction

Delirium, an acute disorder of attention and cognition, is a common and serious complication in hospitalised older adults. Prevalence estimates range from 10 to 50% across hospital settings [1–3], with approximately one in four older inpatients affected [4]. Several factors predispose individuals to delirium, particularly advancing age and preexisting cognitive impairment. Accordingly, delirium and dementia often co-occur in older adults [5, 6], and nearly half of hospitalised patients with dementia experience delirium superimposed on dementia (DSD) [7, 8].

Delirium is associated with adverse outcomes, including long-term cognitive decline, institutionalisation and death [9, 10]. Elevated mortality has been described in both cognitively impaired and cognitively unimpaired individuals with delirium [11, 12], although the magnitude of risk varies across hospital settings [13–23]. Individuals with dementia are at greater baseline risk for delirium [5]. However, whether delirium confers a worse mortality risk specifically among those with dementia remains uncertain. Conceptually, delirium represents an acute insult occurring against a background of cognitive reserve: individuals with lower reserve may be more vulnerable, such that relatively minor precipitating factors trigger delirium. At the same time, those with higher baseline cognition may require a more substantial inciting stimulus to develop delirium [24–26]. Notably, poor outcomes after delirium are observed across diverse aetiologies and persist even when stratified by underlying causes [25].

Despite these risks and uncertainties, few studies have developed risk-stratification approaches to target delirium prevention and management. Moreover, although dementia and delirium frequently co-occur, baseline cognitive status is not usually available at hospital admission. Many older adults with dementia are undiagnosed or unaware of their diagnosis [27], and this challenge may be greater in lower- and middle-income countries [28–30]. These limitations have constrained the understanding of how dementia severity influences delirium outcomes. Therefore, in this multicentre cohort study, we aimed to explore the association between delirium and 90-day mortality and to examine the specific influence of baseline cognitive status on this relationship, which is not well established.

## Methods

The research ethics committees of all participating institutions approved the protocol, and written informed consent was obtained from each patient or from a surrogate when capacity was lacking. Capacity to provide informed consent was determined by the attending physician using a clinical evaluation of the patient’s ability to understand the study’s purpose, risks and benefits, and to communicate a reasoned decision. An authorised representative (e.g. next of kin) provided consent if the patient lacked this capacity (e.g. due to delirium, severe cognitive impairment or reduced level of consciousness). The study was included in a national registry of clinical trials and adhered to the Strengthening the Reporting of Observational Studies in Epidemiology (STROBE) guidelines [31]. Detailed information about the study protocol can be found elsewhere [32].

### Study design and population

We conducted an international, multicentre, prospective cohort study of older adults receiving routine inpatient care at 43 hospitals (38 in Brazil, 1 in Angola, 1 in Chile, 2 in Colombia and 1 in Portugal). Consecutive patients aged ≥65 years admitted under geriatric teams between June 2022 and December 2023 were screened within 48 hours of hospitalisation for eligibility (Appendix 1). Exclusion criteria were terminal illness [Clinical Frailty Scale (CFS) score of 9] [33] and hospital stays <48 hours. Study procedures were standardised across centres.

### Delirium measurement

Within 48 hours of admission, trained raters (registered nurses and physicians) completed a structured assessment that included demographics, medical history, a comprehensive geriatric assessment and a physical examination. Delirium was identified using the Confusion Assessment Method (CAM, short form) [34]. The trained raters conducted the initial delirium assessment at study intake, with daily reassessments performed throughout the hospitalisation, generally during morning rounds, although exact timing could vary by site. The CAM was applied as follows: the features of acute onset/fluctuation were determined using collateral information from care partners or nurses and chart review; inattention was assessed at the bedside via observation and the vigilance ‘A’ test; disorganised thinking was evaluated based on conversational coherence and responses to brief probe questions; and altered level of consciousness was categorised as alert, vigilant, lethargic, stuporous or comatose. The bedside cognitive testing also included the 10-point Cognitive Screener (10-CS), which assesses temporal orientation, three-word recall and semantic fluency (animal naming) [35]. At discharge, delirium status was consolidated to capture both prevalent and incident episodes. Routine delirium screening practices varied across hospitals (the study CAM assessments were performed as part of the research protocol).

### Outcome

The primary outcome was all-cause mortality within 90 days of admission. Participants who were alive at the end of the 90-day follow-up period were right-censored at that time (i.e. treated as event-free up to that time point). Vital status was ascertained through structured telephone interviews at 30 and 90 days, conducted by trained investigators blinded to baseline assessments. If telephone contact was unsuccessful after three attempts, hospital records and public death registries were reviewed. For participants without a confirmed death record, follow-up was right-censored on the last date they were verified to be alive. Thirty-day mortality was analysed as a secondary outcome to characterise short-term risk.

### Effect modifier

Baseline cognitive status was assessed using the global score of the Clinical Dementia Rating (CDR), which stages cognitive and functional performance across memory, orientation, judgement and problem-solving, community affairs, home and hobbies and personal care [36]. The CDR classifies individuals as no dementia (CDR = 0), questionable dementia (CDR = 0.5), mild (CDR = 1), moderate (CDR = 2) or severe (CDR = 3) dementia. The instrument demonstrated 92% sensitivity and 94% specificity for detecting dementia (CDR ≥ 1), using a neurologist adjudication based on DSM-III-R criteria as the clinical reference standard [36]. This level of discrimination exceeds what is typically achievable using electronic medical record data alone. In addition, it is validated for Alzheimer’s disease and related dementias and for use in multicentre studies [36–38]. In this study, we employed the informant-based version, which has been previously validated in this population [39]. This version preserves the original structure and scoring rules while relying solely on knowledgeable informant reports, and showed excellent diagnostic accuracy for dementia [area under the receiver operating characteristic curve (AUC) = 0.92; 95% confidence interval (95% CI) = 0.86–0.98] [39].

### Key variables

Covariate selection was guided by prior literature [40]. At admission, we recorded sociodemographic characteristics [age, sex, marital status, years of education and self-identified race/ethnicity categorised as White, Black or Other (including East Asian and Indigenous)] [41]; vital signs and level of consciousness; and the admission diagnoses. Illness severity was measured using the National Early Warning Score 2 (NEWS-2), which incorporates respiratory rate, oxygen saturation, systolic blood pressure, pulse rate, level of consciousness or confusion and temperature [42]. Disease burden was captured by the Charlson Comorbidity Index (CCI) [43]. We also recorded the number of chronic medications used before admission. At discharge, hospital factors were abstracted, including intensive care unit (ICU) utilisation, length of stay and in-hospital complications. Complications included thrombotic events, weight loss, falls, nosocomial infection, pressure ulcers, functional decline in activities of daily living, prolonged stay (≥14 days) and in-hospital mortality.

### Statistical analysis

Continuous variables were summarised as means with standard deviations (SDs) if normally distributed or medians with interquartile ranges (IQRs) if skewed; categorical variables were summarised as percentages. Groups were defined by the presence or absence of delirium at any time during hospitalisation, and subgroups were defined by CDR category [44]. Between-group comparisons used independent t-tests or Mann–Whitney tests for continuous variables and chi-squared tests for categorical variables.

We estimated survival with Kaplan–Meier methods and compared curves using the log-rank test. To evaluate the association between delirium and all-cause mortality within 90 days of admission, we fit mixed-effects Weibull proportional hazards models with delirium as the primary exposure. We examined effect modification by CDR and generated CDR-stratified survival curves to illustrate adjusted risks across CDR strata. A base model (Model 1) included random intercepts to account for clustering at the state/province and study centre levels. Model 2 additionally adjusted for sociodemographic factors (age, sex, race/ethnicity, education and marital status). Model 3 further adjusted for clinical and hospital-related factors (CDR global score, CCI, number of regularly used medications, modified NEWS-2 excluding the consciousness item, ICU admission and length of stay). This sequential approach was used to characterise the contribution of each set of variables.

Because most prior studies focus on the prognostic impact of prevalent delirium, we conducted subgroup analyses restricted to incident delirium by excluding patients with delirium identified within 48 hours of admission. Analyses were performed in Stata 18 (StataCorp, College Station, TX). Two-sided *P*-values <.05 were considered statistically significant. As fewer than 2% of key variables were missing, we used complete-case analysis.

## Results

Of the 2556 patients enrolled (Appendix 1), 957 (37%) experienced delirium during hospitalisation. The mean age at baseline was 79 years (SD 9); patients with delirium were older than those without delirium [82 (SD 9) vs. 77 (SD 8) years]. Overall, 1437 (56%) were female, with no sex difference by delirium status. Median total years of formal education was 5 years (IQR 3–9), with no difference by delirium status. Baseline cognition comprised 30% with no dementia (CDR 0), 38% with questionable dementia (CDR 0.5), 14% with mild dementia (CDR 1) and 18% with moderate to severe dementia (CDR 2–3). Additional baseline clinical characteristics are reported in Table 1. The majority of participants (*n* = 2390, 94%) were recruited from Brazilian hospitals; 166 participants were from Angola, Chile, Colombia and Portugal (Appendix 2). To evaluate potential heterogeneity by country, we compared patients from Brazil with those from other countries (Appendix 2); Brazilian participants were younger, had higher comorbidity burden and more frequently experienced delirium and dementia.

**Table 1:** Characteristics at admission by delirium during hospitalisation

	Total	No Delirium	Delirium	*P*-value
Variables	(*n* = 2556)	(*n* = 1599)	(*n* = 957)	
***Cognitive measures***				
Clinical Dementia Rating (CDR), *n* (%)				<.001
0: No dementia	765 (30)	639 (40)	126 (13)	
0.5: Questionable dementia	976 (38)	710 (44)	266 (28)	
1: Mild dementia	350 (14)	142 (9)	208 (22)	
2–3: Moderate to severe dementia	465 (18)	108 (7)	357 (37)	
Previous dementia diagnosis, *n* (%)	544 (21)	151 (9)	393 (41)	<.001
Altered level of consciousness, *n* (%)	1374 (54)	559 (35)	815 (85)	<.001
***Sociodemographic characteristics***				
Age (years), mean (SD)	79.2 (9)	77.3 (8)	82.4 (9)	<.001
Female sex, *n* (%)	1437 (56)	889 (56)	548 (57)	.41
Race/ethnicity, *n* (%)				.002
White	1349 (53)	810 (51)	539 (56)	
Black	1139 (45)	753 (47)	386 (40)	
Other	68 (3)	36 (2)	32 (3)	
Education (years), median (IQR)	5 (3, 9)	5 (3, 9)	4 (3, 10)	.11
Married or living with a partner, *n* (%)	1104 (43)	747 (46.7)	357 (37.3)	<.001
***Clinical measures***				
Charlson Comorbidity Index, median (IQR)	2 (1, 5)	2 (1, 4)	2 (1, 5)	.004
Number of chronic medications, median (IQR)	6 (3, 8)	5 (3, 8)	6 (3, 9)	.017
Clinical Frailty Scale (CFS), median (IQR)	5 (3, 7)	4 (3, 6)	6 (5, 7)	<.001
Frailty status (CFS ≥ 5), *n* (%)	1488 (58)	732 (46)	756 (79)	<.001
Acute illness severity (NEWS-2), median (IQR)	4 (2, 6)	3 (1, 5)	5 (4, 7)	<.001
***Hospital-related factors***				
Presence of a hospital companion, *n* (%)				<.001
Always	2013 (79)	1196 (75)	817 (85)	
Sometimes	291 (11)	203 (13)	88 (9)	
Rarely or never	252 (10)	200 (13)	52 (5)	
Intensive care unit (ICU) admission, *n* (%)	408 (16)	209 (13)	199 (21)	<.001
Length of hospital stay (days), median (IQR)	8 (5, 16)	8 (4, 14)	10 (6, 19)	<.001

Abbreviations: IQR, interquartile range; SD, standard deviation; NEWS-2, National Early Warning Score 2.

Values are measured at admission unless otherwise indicated. Percentages are column percentages within delirium strata for categorical variables. Group comparisons used the chi-squared test for categorical variables and the independent t-test or Mann–Whitney test for continuous variables, as appropriate. Altered level of consciousness was assessed at admission and categorised as normal (alert) versus altered (vigilant, lethargic, stuporous or comatose). Other race/ethnicity includes East Asian, Indigenous and not declared. Presence of a hospital companion was recorded at discharge and categorised by time coverage during the hospitalisation. All percentages were rounded to the nearest integer.

Delirium occurred more frequently in those with worsening baseline cognitive status: 126/765 (16%) in CDR 0, 266/976 (27%) in CDR 0.5, 208/350 (59%) in CDR 1 and 357/465 (77%) in CDR 2–3 (*P* < .001). Patients with delirium had a longer length of stay than those without delirium (median 10 vs. 8 days). ICU admission was more frequent among patients with delirium, and their median number of chronic medications was higher (6 vs. 5). In-hospital adverse events were also more common with delirium (Appendix 3). Nosocomial infection was about twice as common in patients with delirium, and clinically meaningful weight loss occurred considerably more frequently. Pressure ulcers, while uncommon overall, were approximately four times more likely to develop. New dependence in activities of daily living showed a moderate increase. Falls were only slightly more frequent, and thrombotic events did not differ between groups.

**Figure 1:**
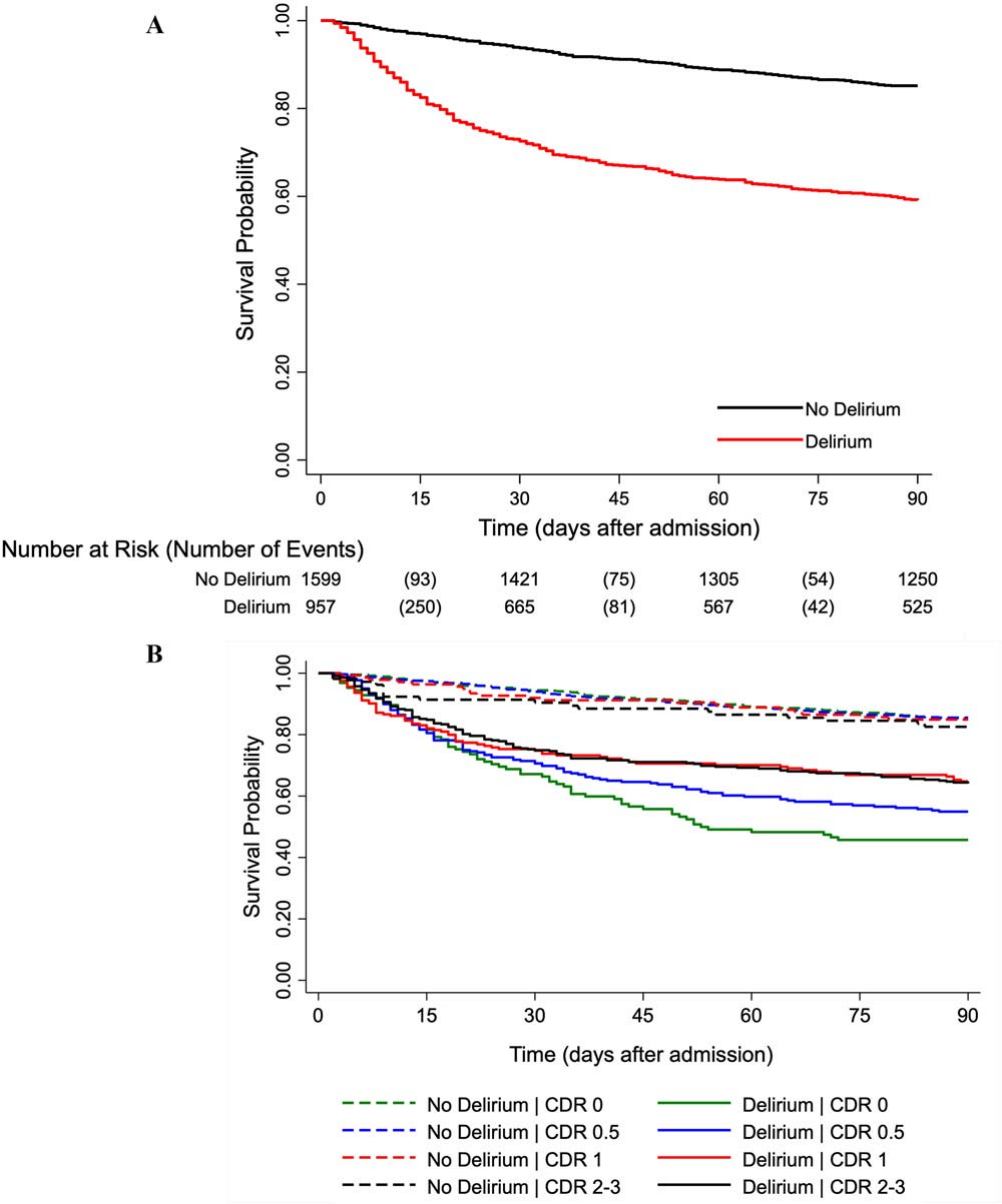
Two Kaplan–Meier plots of survival over 90 days after hospital admission. Panel A compares patients with and without delirium. Survival declines in both groups, but the curve for patients with delirium drops earlier and remains consistently below the curve for patients without delirium throughout follow-up; by day 90, survival is approximately 0.60 in the delirium group and 0.85 in the no-delirium group. Unadjusted survival differed between patients with and without delirium (log-rank χ2(1) = 237.1; *P* < .001). Panel B shows separate survival curves for patients with and without delirium within baseline Clinical Dementia Rating categories 0, 0.5, 1, and 2–3. In every CDR category, survival is lower for patients with delirium than for those without delirium. The separation between delirium and no-delirium curves is greatest in CDR 0 and CDR 0.5 and smaller in CDR 1 and CDR 2–3. Within each CDR stratum, unadjusted survival was lower in patients with delirium than in those without delirium (log-rank *P* ≤ .001 for all comparisons).

Unadjusted Kaplan–Meier estimates showed lower survival rates at 30 and 90 days among patients with delirium (Figure 1A). In mixed-effects Weibull models, delirium was associated with higher 90-day mortality in the base model (HR = 3.93, 95% CI = 3.31–4.67) and after full adjustment (HR = 3.45, 95% CI = 2.83–4.20) (Table 2); country-stratified results were similar (Appendix 4). The association between delirium and mortality attenuated with increasing dementia severity, reflected by stratum-specific interaction terms relative to CDR 0: HRs of 0.82 (95% CI = 0.53–1.25; *P* = .35) for CDR 0.5, 0.57 (95% CI = 0.31–1.04; *P* = .07) for CDR 1 and 0.53 (95% CI = 0.29–0.98; *P* = .04) for CDR 2–3 (Table 3). Within strata, 90-day mortality was 54% with delirium vs. 15% without in CDR 0 (adjusted HR 4.40, 95% CI = 3.15–6.16) and 36% vs. 17% in CDR 2–3 (adjusted HR 2.22, 95% CI = 1.34–3.66), with intermediate strata directionally consistent (Figure 1B; Table 3). Vital status at 90 days was unknown for 186 participants (7.3%). These individuals were last known to be alive a median of 10 days after admission (IQR = 4–33; range 2–62) and were censored at their last contact in the survival analyses.

**Table 2:** Association between delirium during hospitalisation and mortality within 90 days of admission

	*N* died/*N* total (%)	Hazard ratio (95% confidence interval)		
*Outcomes*		Model 1: Base model	Model 2: Base model + sociodemographic factors	Model 3: Fully adjusted model
**30-day mortality**				
Delirium				
No	95/1599 (6)	(reference)	(reference)	(reference)
Yes	254/957 (27)	5.53 (4.34–7.05)	5.15 (4.02–6.60)	5.05 (3.84–6.66)
**90-day mortality**				
Delirium				
No	222/1599 (15)	(reference)	(reference)	(reference)
Yes	374/957 (41)	3.93 (3.31–4.67)	3.74 (3.13–4.47)	3.45 (2.83–4.20)

Estimates are hazard ratios (95% CI) from mixed-effects Weibull proportional hazards models.

Model 1: Base model with random intercepts to account for clustering at the state/province and study centre levels.

Model 2: Model 1 + sociodemographic factors (age, sex, race/ethnicity, education and marital status).

Model 3: Model 2 + CDR global score, CCI, number of regularly used medications, modified NEWS-2 (excluding the consciousness item) and hospital-related factors (intensive care unit admission and length of stay).

Percentages are cumulative mortality within each delirium cell; all percentages were rounded to the nearest integer.

**Table 3:** Effect of baseline cognitive impairment on the association between delirium and 90-day mortality among hospitalised older adults

	No Delirium		Delirium		HR (95% CI) for delirium vs. no delirium within CDR stratum
CDR category	*N* died/*N* total (%)	HR (95% CI)	*N* died/*N* total (%)	HR (95% CI)	
**0: No dementia**	88/639 (15)	1.00 (reference)	67/126 (54)	4.40 (3.15–6.16)	4.40 (3.15–6.16)
**0.5: Questionable dementia**	97/710 (15)	1.04 (0.77–1.39)	114/266 (45)	3.74 (2.78–5.02)	3.60 (2.72–4.76)
**1: Mild dementia**	20/142 (15)	1.23 (0.75–2.03)	69/208 (35)	3.10 (2.21–4.36)	2.52 (1.52–4.17)
**2–3: Moderate to severe dementia**	18/108 (17)	1.61 (0.95–2.72)	123/357 (36)	3.56 (2.62–4.84)	2.22 (1.34–3.66)

Abbreviations: CDR, Clinical Dementia Rating; HR, hazard ratio; 95% CI, 95% confidence interval.

Estimates were obtained from mixed-effects Weibull proportional hazards models with random intercepts for state/province and study centre, adjusted for age, sex, race/ethnicity, education, marital status, CCI, number of regularly used medications, modified NEWS-2 excluding the consciousness item, ICU admission and length of stay. Interaction terms compared each CDR stratum with CDR 0 for the delirium effect on the multiplicative scale, with ratio-of-HRs and *P*-values as follows: CDR 0.5, 0.82 (0.53–1.25), *P* = .35; CDR 1, 0.57 (0.31–1.04), *P* = .07; CDR 2–3, 0.53 (0.29–0.98), *P* = .04.

Percentages are cumulative mortality by 90 days within each CDR-delirium cell; all percentages were rounded to the nearest integer.

Finally, in sensitivity analyses excluding delirium identified within 48 hours of admission, incident delirium remained significantly associated with increased 30- and 90-day mortality (Appendix 5).

## Discussion

In this multicentre prospective cohort across 43 hospitals in 5 countries, nearly 4 in 10 older adults experienced delirium during hospitalisation. The prevalence of delirium varied substantially by baseline cognition, from 16% in those without dementia to 77% in those with moderate to severe dementia. Delirium was strongly associated with 90-day mortality, and this association persisted after adjustment for sociodemographic, clinical and hospital-related factors. Importantly, the magnitude of risk differed by baseline cognition: among patients with no dementia, the relative hazard was approximately four-fold, while among those with moderate to severe dementia, it was about two-fold. Thus, although delirium was more frequent in dementia, its relative prognostic impact was greatest in patients with normal baseline cognition.

Although the association between delirium and mortality is well established, modification by baseline cognition has been less clearly characterised. Evidence from population-based, hospital-based and large prospective cohorts points in the same direction, with higher mortality when delirium occurs among people with better baseline cognition or in the absence of dementia, while dementia itself contributes more to medium- and long-term risk [45–47]. Other cohorts have reported a more substantial association between delirium and mortality among those without dementia, with very low long-term survival when delirium developed in people without dementia [15, 48]. A decade-long UK study reached similar conclusions when delirium was not accompanied by dementia [49]. Together, these studies point toward a greater association between delirium and mortality in patients with no dementia. Even so, most relied on chart-based or nonstandard cognitive classifications, varied case-finding for delirium and rarely quantified stratum-specific hazards across validated dementia stages.

Our study addresses these gaps by quantifying how the prognostic association between delirium and mortality varies across rigorously measured baseline cognitive strata. Unlike studies that inferred dementia from chart diagnoses or proxy indicators, we staged cognition using the CDR and identified delirium with standardised CAM assessments. The multisite design, consistent estimates across multivariable models, and coherent gradient across CDR strata support the inference that delirium’s prognostic weight is greatest among cognitively unimpaired older adults.

Patients with dementia have higher baseline mortality risk, and greater dementia severity is typically associated with higher mortality [50, 51]. In contrast, when focusing on the excess risk associated with delirium, we observed the strongest relative association in patients without dementia. These findings highlight the need for intervention trials to determine if modifying delirium risk improves outcomes across cognitive strata. Patients at the highest relative risk of death following delirium may be those in whom delirium is least anticipated. Although enhanced detection in patients with dementia remains essential [52], our results suggest equal vigilance is warranted for those without known cognitive impairment. In practical terms, delirium in an older inpatient without recognised cognitive impairment should be treated as a high-acuity safety signal, prompting timely evaluation for reversible precipitants and closer clinical monitoring (Box 1) [53, 54]. Moreover, because baseline cognitive status is often unavailable or unreliable at admission and dementia is commonly under-recognised, delirium prevention and detection strategies should be implemented broadly rather than restricted to those with documented dementia.

**Box 1:** Practical implications: delirium as a hospital-wide priority across baseline cognition.

**A universal imperative for detection**	Consider systematic daily screening for all older inpatients using validated tools (e.g. CAM, 4AT, NuDESC). Do not rely on clinical suspicion alone, which risks missing the syndrome where it is least expected but most prognostically significant.
**Considering delirium when cognition is reportedly normal**	Treat delirium in a patient thought to be cognitively intact as a high-acuity clinical sign: it often signals a severe physiological insult and warrants urgent review. Engage families and care partners; their report of an acute mental change is highly informative and should prompt timely action. Recognise that these patients may be at particularly high short-term risk, benefiting from enhanced monitoring, a comprehensive geriatric assessment and meticulous planning for discharge and recovery.
**Managing delirium when dementia is known or suspected**	Maintain high vigilance; adherence to multicomponent, non-pharmacological prevention strategies (e.g. reorientation, sleep hygiene and mobility) is essential. Acknowledge prognostic nuance: while delirium adds excess risk, the patient’s underlying dementia stage can modify the association with adverse outcomes. Ensure management addresses both the acute delirium and the person’s broader cognitive care needs.
**Planning for discharge, recovery and follow-up**	Delirium and the patient's cognitive status should be explicitly documented in discharge summaries and communicated to the patient, family and primary care team. Schedule early post-discharge review to monitor cognitive recovery, medication safety and functional status. Provide anticipatory guidance on delirium course and the potential long-term implications.
**System-level considerations**	Reframe the rationale for delirium programmes as a universal patient-safety imperative, not solely an issue for patients with known high vulnerability. Build scalable detection capacity by training frontline staff in brief assessments and informant-based tools that include documentation of baseline cognition.

Several mechanisms may explain the larger relative association of delirium with mortality among cognitively unimpaired individuals. Delirium in these patients may require a higher physiological or environmental threshold, implying exposure to more severe hospital stressors despite adjustment for illness severity. Conversely, among patients with dementia, delirium may represent a less severe additional insult given lower cognitive reserve. Prior work from a Brazilian hospital cohort reported high in-hospital mortality in DSD [47]. In our study, time-to-death was measured from admission, which reduces (but does not eliminate) the potential for immortal time bias. Of all deaths, 58% occurred in the hospital; among these in-hospital deaths, 24% were in the no dementia group (CDR 0), 34% in questionable dementia (CDR 0.5), 16% in mild dementia (CDR 1) and 26% in moderate to severe dementia (CDR 2–3). A similar pattern was observed for 90-day mortality after discharge, with 29% for no dementia, 37% for questionable dementia, 14% for mild dementia and 20% for moderate to severe dementia. The higher mortality observed among cognitively unimpaired participants could reflect residual confounding by factors not captured in this analysis (e.g. cancer, immobility, hypoalbuminemia, renal dysfunction or heart failure) [40], even after adjustment for comorbidities using the CCI.

### Limitations and strengths

Our study had limitations. Centre participation was based on interest rather than population sampling, and most participants were from Brazil, which may affect generalisability. Screening capacity varied across sites, resulting in some eligible admissions not being assessed. Given the multicentre design, rater variability is possible; for example, CDR assessments may have differed across sites [38]. In addition, we modelled delirium as a binary exposure and did not assess severity. Prior work among patients with Alzheimer’s disease and related dementias has shown a trend toward higher 1-month mortality with greater delirium severity [55]. We also did not examine delirium duration or psychomotor subtype, factors that may further influence outcomes [56–58].

The study also had notable strengths. The cohort was large (>2500 older adults) and spanned more than 40 hospitals in five countries, many in low- and middle-income settings, with participants who had fewer years of formal education and diverse racial and ethnic backgrounds compared with cohorts from higher-income countries. This real-world design addresses geographic and socioeconomic gaps in the delirium literature. Even so, the high absolute burden of delirium observed in LMIC hospitals reinforces the urgency of standardised delirium and dementia screening. Our work points to the research feasibility of implementing CDR interviews, a validated tool with high sensitivity and specificity [36, 39], and CAM screening across numerous LMIC hospitals, supporting further investigation into the scalability of standardised delirium detection in clinical practice.

## Conclusion

In summary, delirium during hospitalisation was associated with substantially higher 90-day mortality in this multicentre cohort, with the strongest relative association observed among patients without dementia. These findings challenge a common clinical assumption that delirium poses the greatest threat to patients with preexisting cognitive impairment. Instead, our data demonstrate that delirium independently predicts short-term mortality across all cognitive states, with particularly striking prognostic implications in patients previously presumed to be at lower risk. This has immediate clinical implications. Delirium detection should be a priority for all hospitalised older adults and not limited to those with known dementia. Clinicians should approach delirium with the understanding that it carries substantial short-term mortality risk and may signal severe underlying illness, particularly when it occurs in patients without dementia.

## Supplementary Material

aa-25-3334-File002_afag081

## Data Availability

Deidentified individual participant data (with data dictionary and analysis code) will be made available upon reasonable request to the corresponding author beginning 12 months after publication for researchers with methodologically sound proposals and a signed data use agreement; requests will be reviewed by the CHANGE Steering Committee.
