# Supplementary material for: Frequent in dementia, deadliest without it: delirium and mortality in hospitalised older adults
Source: Age Ageing. 2026 Apr 9;55(4):afag081. doi: 10.1093/ageing/afag081 (PMC13070384; doi:10.1093/ageing/afag081)
Supplement: aa-25-3334-File002_afag081 [file aa-25-3334-file002_afag081.docx]

**SUPPLEMENTARY DATA**

**Appendix 1.** Flowchart of the study participants (Figure)

**Appendix 2.** Characteristics of the study population stratified by country (Table)

**Appendix 3.** In‑hospital adverse events according to delirium status among older adults (N=2,556) (Table)

**Appendix 4.** Association between delirium in the hospital and 90‑day mortality stratified by country (Table)

**Appendix 5.** Delirium type analyses (Tables)

A. Distribution of prevalent and incident delirium rates by CDR categories (CDR 0, 0.5, 1, 2–3).

B. Association between delirium type (prevalent vs. incident) and 30‑day and 90‑day mortality with hazard ratios (95% CIs) from Models 1–3.

**Appendix 6.** CHANGE Study group collaborators.

**Appendix 1.** Flowchart of the study participants.


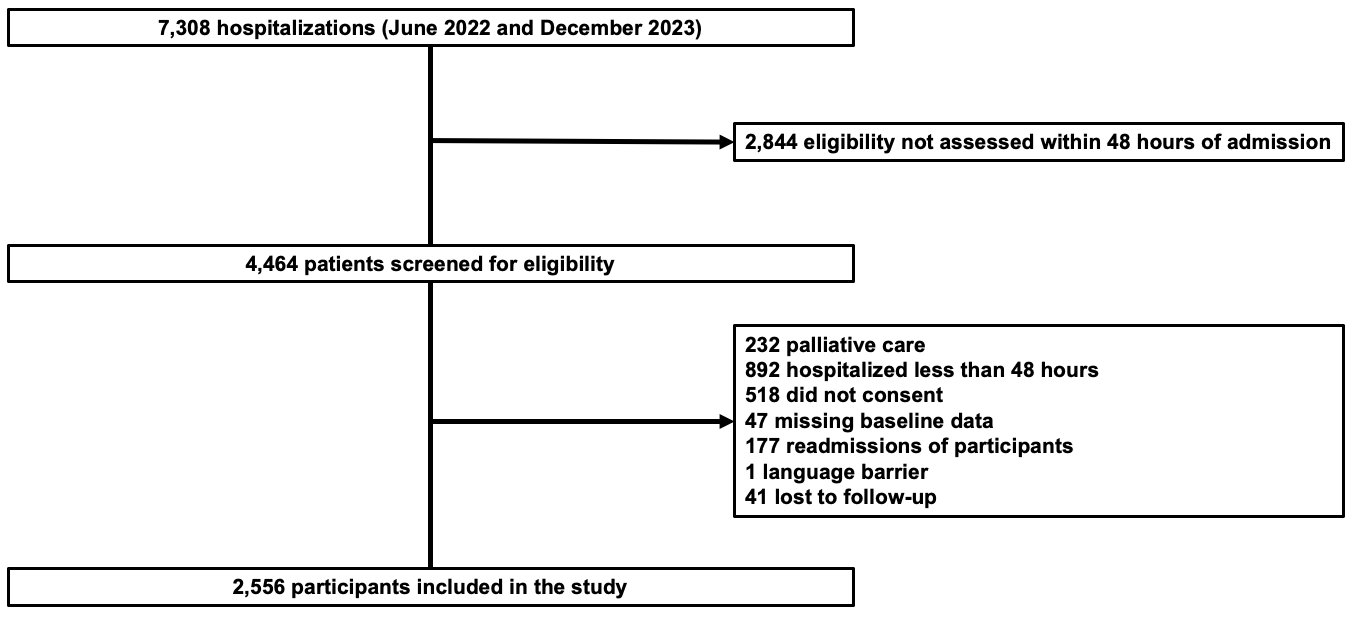


Flow of participant screening and enrolment for the multicentre cohort. Consecutive admissions of adults aged ≥65 years under geriatric teams were screened within 48 hours of hospitalisation. Exclusions comprised terminal illness, defined as a Clinical Frailty Scale score of 9, and hospital stays <48 hours. The final analytic sample included 2,556 patients. Country distribution is provided in Appendix 2.

**Appendix 2.** Characteristics of the study population stratified by country.

|  | **Other countries** | **Brazil** | ***p*-value** |
| --- | --- | --- | --- |
| **Variables** | **(N = 166)** | **(N = 2,390)** |  |
| ***Cognitive measures*** |  |  |  |
| Delirium in the hospital, N (%) | 48 (29) | 909 (38) | 0.01 |
| Clinical Dementia Rating (CDR), N (%) |  |  | 0.001 |
| 0: No dementia | 51 (31) | 714 (30) |  |
| 0.5: Questionable dementia | 63 (38.0) | 913 (38) |  |
| 1: Mild dementia | 36 (22) | 314 (13) |  |
| 2-3: Moderate to severe dementia | 16 (10) | 449 (19) |  |
| Previous dementia diagnosis, N (%) | 20 (12) | 524 (22) | 0.003 |
| Altered level of consciousness, N (%) | 81 (49) | 1293 (54) | 0.18 |
| ***Sociodemographic characteristics*** |  |  |  |
| Age (years), mean (SD) | 82.3 (9) | 79.0 (9) | <0.001 |
| Female sex, N (%) | 95 (57) | 1342 (56) | 0.79 |
| Race/ethnicity, N (%) |  |  | <0.001 |
| White | 42 (25) | 1307 (55) |  |
| Black | 116 (70) | 1023 (43) |  |
| Other | 8 (5) | 60 (2.5) |  |
| Education (years), median (IQR) | 4 (2, 8) | 5 (3, 9) | 0.005 |
| Married or living with a partner, N (%) | 77 (46) | 1027 (43) | 0.39 |
| ***Clinical measures*** |  |  |  |
| Charlson Comorbidity Index, median (IQR) | 1 (0, 3) | 2 (1, 5) | <0.001 |
| Number of medications in use, median (IQR) | 4 (1, 6) | 6 (3, 9) | <0.001 |
| Acute illness severity (NEWS-2), median (IQR) | 3 (2, 6) | 4 (2, 6) | 0.055 |
| ***Hospital-related factors*** |  |  |  |
| Intensive care unit (ICU) admission, N (%) | 8 (5) | 400 (17) | <0.001 |
| Length of hospital stay (days), median (IQR) | 6 (4, 9) | 9 (5, 16) | 0.001 |

IQR = interquartile range; SD = standard deviation; NEWS-2 = National Early Warning Score 2.

Values are measured at admission unless otherwise stated. Group comparisons used the chi‑squared test for categorical variables and the independent t‑test or the Mann–Whitney test for continuous variables, as appropriate. All percentages were rounded to the nearest integer.

**Appendix 3.** In-hospital adverse events according to delirium status among older adults (N = 2,556).

|  | **Total** | **No Delirium** | **Delirium** | ***p*-value** |
| --- | --- | --- | --- | --- |
| ***In-hospital events, N (%)*** | **(N = 2,556)** | **(N = 1,599)** | **(N = 957)** |  |
| Thrombotic events | 52 (2) | 29 (2) | 23 (2) | 0.31 |
| Weight loss ≥ 5% | 501 (20) | 255 (16) | 246 (26) | <0.001 |
| Fall | 49 (2) | 24 (2) | 25 (3) | 0.047 |
| Nosocomial infection | 605 (24) | 277 (17) | 328 (34) | <0.001 |
| Pressure ulcer | 48 (2) | 10 (1) | 38 (4) | <0.001 |
| Functional decline (ADLs) | 1,486 (58) | 824 (52) | 662 (69) | <0.001 |
| Prolonged stay (≥ 14 days) | 781 (31) | 426 (27) | 355 (37) | <0.001 |
| Mortality | 344 (14) | 82 (5) | 262 (27) | <0.001 |

ADLs = basic activities of daily living (bathing, dressing, toileting, transferring, eating, and continence).

Events were abstracted at discharge and refer to occurrences during the hospitalisation. Thrombotic events included deep‑vein thrombosis and pulmonary embolism. Nosocomial infections included pulmonary, urinary tract, bloodstream, surgical site, and catheter‑related infections. Weight loss was defined as ≥5% reduction from admission weight. Functional decline in ADLs was defined as new dependence compared with 2–4 weeks before admission; 229 patients who were fully dependent at baseline were excluded from this calculation. Prolonged stay was defined as ≥14 days (upper tertile). All percentages were rounded to the nearest integer.

**Appendix 4.** Association between delirium in the hospital and 90-day mortality stratified by country

|  | **N died / N total (%)** | **Hazard ratio (95% confidence interval)** | | |
| --- | --- | --- | --- | --- |
| **Outcomes** |  | **Model 1: Base model** | **Model 2: Base model + sociodemographic factors** | **Model 3: Fully adjusted model** |
| ***Brazil (N = 2,390)*** | |  |  |  |
| Delirium |  |  |  |  |
| No | 211/1,481 (15) | (reference) | (reference) | (reference) |
| Yes | 358/909 (41) | 3.93 (3.29–4.69) | 3.71 (3.10–4.45) | 3.50 (2.86–4.28) |
| ***Other countries (N = 166)*** | |  |  |  |
| Delirium |  |  |  |  |
| No | 11/118 (10) | (reference) | (reference) | (reference) |
| Yes | 16/48 (35) | 4.31 (1.90–9.79) | 5.35 (2.05–14.0) | 3.38 (1.22–9.38) |

Estimates are hazard ratios (95%CI) from mixed‑effects Weibull proportional hazards models for 90‑day mortality

Model 1: Base model with random intercepts to account for clustering at the state/province and study centre levels.

Model 2: Model 1 + sociodemographic factors (age, sex, race/ethnicity, education, and marital status).

Model 3: Model 2 + Clinical Dementia Rating (CDR) global score, Charlson Comorbidity Index, number of regularly used medications, modified National Early Warning Score 2 (NEWS-2, excluding the consciousness item), and hospital-related factors (intensive care unit admission and length of stay).

Percentages are cumulative mortality within each delirium cell; all percentages were rounded to the nearest integer.

**Appendix 5**

1. Distribution of prevalent and incident delirium rates by CDR category

| **Delirium type** | **CDR=0 (n=765)** | **CDR=0.5 (n=976)** | **CDR=1**  **(n=350)** | **CRD=2-3 (n=465)** |
| --- | --- | --- | --- | --- |
| No delirium | 639 (84%) | 710 (73%) | 142 (41%) | 108 (23%) |
| Prevalent delirium | 56 (7%) | 126 (13%) | 130 (37%) | 241 (52%) |
| Incident delirium | 70 (9%) | 140 (14%) | 78 (22%) | 116 (25%) |

Percentages are within CDR columns and were rounded to the nearest integer. Prevalent delirium was identified within 48 hours of admission. Incident delirium was identified after 48 hours.

**B.** Association between delirium type and mortality (N = 2,556)

|  | **N died / N total (%)** | **Hazard ratio (95% confidence interval)** | | |
| --- | --- | --- | --- | --- |
| ***Outcomes*** |  | **Model 1: Base model** | **Model 2: Base model + sociodemographic factors** | **Model 3: Fully adjusted model** |
| **30-day mortality** |  |  |  |  |
| Delirium |  |  |  |  |
| No | 95/1,599 (6) | (reference) | (reference) | (reference) |
| Prevalent | 160/553 (30) | 6.20 (4.78–8.06) | 5.71 (4.36–7.48) | 5.19 (3.84–7.01) |
| Incident | 94/404 (24) | 4.82 (3.59–6.46) | 4.62 (3.44–6.22) | 5.11 (3.72–7.02) |
| **90-day mortality** |  |  |  |  |
| Delirium |  |  |  |  |
| No | 222/1,599 (15) | (reference) | (reference) | (reference) |
| Prevalent | 221/553 (42) | 4.10 (3.38–4.98) | 3.87 (3.17–4.72) | 3.52 (2.82–4.41) |
| Incident | 153/404 (39) | 3.76 (3.04–4.66) | 3.65 (2.94–4.53) | 3.44 (2.74–4.33) |

Estimates are hazard ratios (95%CI) from mixed-effects Weibull proportional hazards models.

Model 1: Base model with random intercepts to account for clustering at the state/province and study centre levels.

Model 2: Model 1 + sociodemographic factors (age, sex, race/ethnicity, education, and marital status).

Model 3: Model 2 + Clinical Dementia Rating (CDR) global score, Charlson Comorbidity Index, number of regularly used medications, modified National Early Warning Score 2 (NEWS-2, excluding the consciousness item), and hospital-related factors (intensive care unit admission and length of stay).

Percentages are cumulative mortality within each delirium cell; all percentages were rounded to the nearest integer.

**Appendix 6.** CHANGE Study Group collaborators.

| ***First Name and Middle Initial(s)** | ***Last Name** | ***Suffix (eg, Jr, III)** | **Academic Degrees** | **Institution** | **Location (city, state/province, country)** |
| --- | --- | --- | --- | --- | --- |
| Gabriel T. | Constantino |  | MD | A. C. Camargo Cancer Center | São Paulo, SP, Brazil |
| Ivan M. | de Oliveira |  | MD | Beneficência Portuguesa de São Paulo | São Paulo, SP, Brazil |
| Danilsa V. | de Sousa |  | MD | Clínica Girassol | Luanda, Angola |
| Diana | Francisco |  | MD | Clínica Girassol | Luanda, Angola |
| Eunice | Andrade |  | MD | Clínica Girassol | Luanda, Angola |
| Nazareth | Neto |  | MD | Clínica Girassol | Luanda, Angola |
| Nidia | Van Dunem |  | MD | Clínica Girassol | Luanda, Angola |
| Kenneth E. | Covinsky |  | MD, MPH | Division of Geriatrics, University of California, San Francisco | San Francisco, CA, USA |
| Beatriz N. | da Cunha |  | MD | Hospital Central da Irmandade da Santa Casa de Misericórdia de São Paulo | São Paulo, SP, Brazil |
| Ewerton | Miyadahira |  | MD | Hospital Central da Irmandade da Santa Casa de Misericórdia de São Paulo | São Paulo, SP, Brazil |
| Gustavo M. | Mil Homens |  | MD | Hospital Central da Irmandade da Santa Casa de Misericórdia de São Paulo | São Paulo, SP, Brazil |
| Lisa L. | Mello |  | MD | Hospital Central da Irmandade da Santa Casa de Misericórdia de São Paulo | São Paulo, SP, Brazil |
| Marcos D. C. | Saraiva |  | MD, PhD | Hospital Central da Irmandade da Santa Casa de Misericórdia de São Paulo | São Paulo, SP, Brazil |
| Mariana M. | Teruya |  | MD | Hospital Central da Irmandade da Santa Casa de Misericórdia de São Paulo | São Paulo, SP, Brazil |
| Mario S. S. | Cabral |  | MD | Hospital Central da Irmandade da Santa Casa de Misericórdia de São Paulo | São Paulo, SP, Brazil |
| Matheus P. | Viola |  | MD | Hospital Central da Irmandade da Santa Casa de Misericórdia de São Paulo | São Paulo, SP, Brazil |
| Milton L. | Gorzoni |  | MD, PhD | Hospital Central da Irmandade da Santa Casa de Misericórdia de São Paulo | São Paulo, SP, Brazil |
| Renato T. | Galvão |  | MD | Hospital Central da Irmandade da Santa Casa de Misericórdia de São Paulo | São Paulo, SP, Brazil |
| Beatriz N. | Nassif |  | MD | Hospital das Clínicas da Faculdade de Medicina da USP | São Paulo, SP, Brazil |
| Dannielle M. | Guimarães |  | MD | Hospital das Clínicas da Faculdade de Medicina da USP | São Paulo, SP, Brazil |
| Ezemir D. | Fernandes | Junior | MD | Hospital das Clínicas da Faculdade de Medicina da USP | São Paulo, SP, Brazil |
| Flavia | Campora |  | MD | Hospital das Clínicas da Faculdade de Medicina da USP | São Paulo, SP, Brazil |
| Flavia A. | de Amorim |  | MD | Hospital das Clínicas da Faculdade de Medicina da USP | São Paulo, SP, Brazil |
| Flavia T. T. | Nakamura |  | MD | Hospital das Clínicas da Faculdade de Medicina da USP | São Paulo, SP, Brazil |
| Jose S. | Cardoso | Neto | MD | Hospital das Clínicas da Faculdade de Medicina da USP | São Paulo, SP, Brazil |
| Julia M. | Menezes |  | MD | Hospital das Clínicas da Faculdade de Medicina da USP | São Paulo, SP, Brazil |
| Luis E. M. | Martins |  | MD | Hospital das Clínicas da Faculdade de Medicina da USP | São Paulo, SP, Brazil |
| Mayara V. | Batista |  | MD | Hospital das Clínicas da Faculdade de Medicina da USP | São Paulo, SP, Brazil |
| Raiza T. | Lira |  | MD | Hospital das Clínicas da Faculdade de Medicina da USP | São Paulo, SP, Brazil |
| Silvio C. | Amorim |  | MD | Hospital das Clínicas da Faculdade de Medicina da USP | São Paulo, SP, Brazil |
| Vitor A. | Fontenelles |  | MD | Hospital das Clínicas da Faculdade de Medicina da USP | São Paulo, SP, Brazil |
| Gabriela M. | Costa |  | - | Hospital das Clínicas da Faculdade de Medicina de Botucatu | Botucatu, SP, Brazil |
| Paulo José F. | Villas Boas |  | MD, PhD, MSc | Hospital das Clínicas da Faculdade de Medicina de Botucatu | Botucatu, SP, Brazil |
| Julio C. | Moriguti |  | MD, PhD | Hospital das Clínicas da Faculdade de Medicina de Ribeirão Preto | Ribeirão Preto, SP, Brazil |
| Nereida K. C. | Lima |  | MD, PhD | Hospital das Clínicas da Faculdade de Medicina de Ribeirão Preto | Ribeirão Preto, SP, Brazil |
| Edgar N. | de Moraes |  | MD, PhD | Hospital das Clínicas da Universidade Federal de Minas Gerais | Belo Horizonte, MG, Brazil |
| Maria Aparecida C. | Bicalho |  | MD, PhD, MSc | Hospital das Clínicas da Universidade Federal de Minas Gerais | Belo Horizonte, MG, Brazil |
| Tatiana C. E. | Pinheiro |  | MD, MS | Hospital das Clínicas da Universidade Federal de Minas Gerais | Belo Horizonte, MG, Brazil |
| Alicia R. M. | Accioly |  | MD, MSc | Hospital das Clínicas da Universidade Federal de Pernambuco | Recife, PE, Brazil |
| Hugo O. D. M. | Gomes |  | MD | Hospital das Clínicas da Universidade Federal de Pernambuco | Recife, PE, Brazil |
| Maria M. V. | Guedes |  | MD, MSc | Hospital das Clínicas da Universidade Federal de Pernambuco | Recife, PE, Brazil |
| Mariana A. | de Luna |  | MD | Hospital das Clínicas da Universidade Federal de Pernambuco | Recife, PE, Brazil |
| Mayara S. | Honorato |  | MD | Hospital das Clínicas da Universidade Federal de Pernambuco | Recife, PE, Brazil |
| Milena B. A. | Silva |  | MD | Hospital das Clínicas da Universidade Federal de Pernambuco | Recife, PE, Brazil |
| Rebeca M. S. | Coelho |  | MD | Hospital das Clínicas da Universidade Federal de Pernambuco | Recife, PE, Brazil |
| Rosana S. | Batista |  | MD | Hospital das Clínicas da Universidade Federal de Pernambuco | Recife, PE, Brazil |
| Andre | Fattori |  | MD, PhD | Hospital de Clínicas - UNICAMP | Campinas, SP, Brazil |
| Estela F. | Vilela |  | MD | Hospital de Clínicas - UNICAMP | Campinas, SP, Brazil |
| Jessica | Valonini |  | MD | Hospital de Clínicas - UNICAMP | Campinas, SP, Brazil |
| Rodolfo A. O. | Nogueira |  | MD | Hospital de Clínicas - UNICAMP | Campinas, SP, Brazil |
| Emilio H. | Moriguchi |  | MD, MSc,PhD | Hospital de Clínicas de Porto Alegre | Porto Alegre, RS, Brazil |
| Francine F. | Klein |  | MSc, PhD | Hospital de Clínicas de Porto Alegre | Porto Alegre, RS, Brazil |
| Renato G. Bandeira | de Mello |  | MD, PhD, MPH | Hospital de Clínicas de Porto Alegre | Porto Alegre, RS, Brazil |
| Juliana J. M. | Teixeira |  | MD, MSc | Hospital de Urgências de Goiás | Goiânia, GO, Brazil |
| Adriana F. | Dutra |  | RN | Hospital do Coração | São Paulo, SP, Brazil |
| Aline T. S. | Santos |  | MD | Hospital do Coração de Natal | Natal, RN, Brazil |
| Beatriz N. A. | Lopes |  | MD | Hospital do Coração de Natal | Natal, RN, Brazil |
| Giovanni G. N. | Santos |  | MD | Hospital do Coração de Natal | Natal, RN, Brazil |
| João G. A. | de Lima |  | - | Hospital do Coração de Natal | Natal, RN, Brazil |
| Juliana C. | de Souza |  | MD | Hospital do Coração de Natal | Natal, RN, Brazil |
| Juliano S. | de Araujo |  | MD | Hospital do Coração de Natal | Natal, RN, Brazil |
| Maria C. T. | Vianna |  | - | Hospital do Coração de Natal | Natal, RN, Brazil |
| Natalia C. | Guedes |  | MD | Hospital do Coração de Natal | Natal, RN, Brazil |
| Rafael V. S. | Barreto |  | - | Hospital do Coração de Natal | Natal, RN, Brazil |
| Raphael A. | Filgueiras |  | - | Hospital do Coração de Natal | Natal, RN, Brazil |
| Rayane L. C. D. | de Medeiros |  | - | Hospital do Coração de Natal | Natal, RN, Brazil |
| Eduardo Marques | da Silva |  | MD | Hospital Emílio Carlos, Centro Universitário Padre Albino | Catanduva, SP, Brazil |
| Brunna S. | Oliveira |  | PharmD | Hospital Geral de Vitória da Conquista | Vitória da Conquista, BA, Brazil |
| Esther S. M. | Melo |  | Psychologist | Hospital Geral de Vitória da Conquista | Vitória da Conquista, BA, Brazil |
| Hellen M. M. | Cardoso |  | Psychologist | Hospital Geral de Vitória da Conquista | Vitória da Conquista, BA, Brazil |
| Iuri C. | Gusmão |  | - | Hospital Geral de Vitória da Conquista | Vitória da Conquista, BA, Brazil |
| Jonatas S. P. | Porto |  | MD | Hospital Geral de Vitória da Conquista | Vitória da Conquista, BA, Brazil |
| Marcio G. G. | de Oliveira |  | PharmD, MSc, PhD | Hospital Geral de Vitória da Conquista | Vitória da Conquista, BA, Brazil |
| Maria E. S. G. | Roberto |  | MD | Hospital Geral de Vitória da Conquista | Vitória da Conquista, BA, Brazil |
| Nara L. F. | Rebouças |  | - | Hospital Geral de Vitória da Conquista | Vitória da Conquista, BA, Brazil |
| Roberta B. | Jauris |  | Psychologist, MSc | Hospital Geral de Vitória da Conquista | Vitória da Conquista, BA, Brazil |
| Tatiane D. C. | Valença |  | PT, MSc, PhD | Hospital Geral de Vitória da Conquista | Vitória da Conquista, BA, Brazil |
| Welma W. C. C. | Amorim |  | MD, PhD, MSc | Hospital Geral de Vitória da Conquista | Vitória da Conquista, BA, Brazil |
| Andrezza M. | Fernandes |  | MD | Hospital Geral Dr. César Cals de Oliveira | Fortaleza, CE, Brazil |
| Carolina M. | Feijo |  | MD | Hospital Geral Dr. César Cals de Oliveira | Fortaleza, CE, Brazil |
| Hellen M. P. | Rocha |  | RN | Hospital Geral Dr. César Cals de Oliveira | Fortaleza, CE, Brazil |
| Ianna L. S. | Braga |  | MD, PhD, MSc | Hospital Geral Dr. César Cals de Oliveira | Fortaleza, CE, Brazil |
| Lara A. | Vieira |  | MD | Hospital Geral Dr. César Cals de Oliveira | Fortaleza, CE, Brazil |
| Lirenna P. | Narciso |  | MD | Hospital Geral Dr. César Cals de Oliveira | Fortaleza, CE, Brazil |
| Luisa B. | Bruno |  | MD | Hospital Geral Dr. César Cals de Oliveira | Fortaleza, CE, Brazil |
| Nadedja L. Q. | Rocha |  | MD | Hospital Geral Dr. César Cals de Oliveira | Fortaleza, CE, Brazil |
| Priscila P. S. | Nogueira |  | MD | Hospital Geral Dr. César Cals de Oliveira | Fortaleza, CE, Brazil |
| Rafael S. B. | Pinheiro |  | MD | Hospital Geral Dr. César Cals de Oliveira | Fortaleza, CE, Brazil |
| Wallena C. | Brito |  | MD | Hospital Geral Dr. César Cals de Oliveira | Fortaleza, CE, Brazil |
| Yngrid Dieguez | Ferreira |  | MD | Hospital Geriátrico e de Convalescentes Dom Pedro II | São Paulo, SP, Brazil |
| Adriana | Alves |  | RN | Hospital Israelita Albert Einstein | São Paulo, SP, Brazil |
| Julia M. | Menezes |  | MD | Hospital Israelita Albert Einstein | São Paulo, SP, Brazil |
| Margarete C. P. | Miralia |  | RN | Hospital Israelita Albert Einstein | São Paulo, SP, Brazil |
| Natascha G. F. | Palmeira |  | PhD | Hospital Israelita Albert Einstein | São Paulo, SP, Brazil |
| Vanessa A. L. | Pires |  | RN | Hospital Israelita Albert Einstein | São Paulo, SP, Brazil |
| Victor J. D. | Melo |  | MD | Hospital Israelita Albert Einstein | São Paulo, SP, Brazil |
| Laiane M. | Dias |  | MD,PhD | Hospital Jean Bitar | Belém,PA, Brazil |
| Alexandra | Malheiro |  | MD, MSc | Hospital Lusíadas Porto | Porto, Portugal |
| Filipe | Basto |  | MD | Hospital Lusíadas Porto | Porto, Portugal |
| Vitor L. | Pintarelli |  | MD, PhD | Hospital Nossa Senhora das Graças | Curitiba, PR, Brazil |
| Fabiola | Sepulveda |  | MD | Hospital Regional de Talca Dr César Garavagno Burotto | Talca, Chile |
| Camila F. | Lima |  | MD | Hospital Santo Antônio - Obras Sociais Irmã Dulce | Salvador, BA, Brazil |
| Dominique K. B. | Silva |  | MD | Hospital Santo Antônio - Obras Sociais Irmã Dulce | Salvador, BA, Brazil |
| Josecy M. S. | Peixoto |  | MS, PhD | Hospital Santo Antônio - Obras Sociais Irmã Dulce | Salvador, BA, Brazil |
| Lucas K. P. | Prado |  | MD, MSc | Hospital Santo Antônio - Obras Sociais Irmã Dulce | Salvador, BA, Brazil |
| Manuela O. C. | Magalhães |  | MS, PhD | Hospital Santo Antônio - Obras Sociais Irmã Dulce | Salvador, BA, Brazil |
| Paula L. | Ferreia |  | MD | Hospital Santo Antônio - Obras Sociais Irmã Dulce | Salvador, BA, Brazil |
| Rísia M. O. | Barreto |  | MD | Hospital Santo Antônio - Obras Sociais Irmã Dulce | Salvador, BA, Brazil |
| Tatiana S. | Moreira |  | MD | Hospital Santo Antônio - Obras Sociais Irmã Dulce | Salvador, BA, Brazil |
| Alayne M. T. D. | Yamada |  | PhD | Hospital São Camilo | São Paulo, SP, Brazil |
| Fabio A. | Bittencourt |  | MD | Hospital São Camilo | São Paulo, SP, Brazil |
| Graziela B. B. | Ivanov |  | MD | Hospital São Camilo | São Paulo, SP, Brazil |
| Mara G. M. | Silveira |  | MD | Hospital São Camilo | São Paulo, SP, Brazil |
| Oberdã G. | Moreira-Filho |  | MD, MSc | Hospital São Camilo | São Paulo, SP, Brazil |
| Debora D. | Casagrande |  | MD | Hospital São José | Criciúma, SC, Brazil |
| Gabriela S. | Keller |  | MD, MSc | Hospital São José | Criciúma, SC, Brazil |
| Thatiana | Dal Toe |  | MD,MS | Hospital São José | Criciúma, SC, Brazil |
| Clineu M. | Almada-Filho |  | MD, PhD, MSc | Hospital São Paulo - UNIFESP | São Paulo, SP, Brazil |
| Eduardo C. | Cruz |  | MD | Hospital São Paulo - UNIFESP | São Paulo, SP, Brazil |
| Lara M. Q. | Araujo |  | MD, PhD, MSc | Hospital São Paulo - UNIFESP | São Paulo, SP, Brazil |
| Natalia I. B. | Garção |  | MD | Hospital São Paulo - UNIFESP | São Paulo, SP, Brazil |
| Perola Q. | de Almeida |  | MD, MSc | Hospital São Paulo - UNIFESP | São Paulo, SP, Brazil |
| Bruna M. | de Carvalho |  | MD | Hospital Sírio Libanês | São Paulo, SP, Brazil |
| Maria E. | Pires |  | MD | Hospital Sírio Libanês | São Paulo, SP, Brazil |
| Michel S. | Dantas |  | RPh | Hospital Sírio Libanês | São Paulo, SP, Brazil |
| Naira H. S. L. | Hojaij |  | MD, PhD | Hospital Sírio Libanês | São Paulo, SP, Brazil |
| Julia F. | Brenny |  | - | Hospital Universitário da Universidade Estadual de Londrina | Londrina, PR, Brazil |
| Pedro H. A. | Silva |  | - | Hospital Universitário da Universidade Estadual de Londrina | Londrina, PR, Brazil |
| Renata M. | Dip |  | MD, PhD, MSc | Hospital Universitário da Universidade Estadual de Londrina | Londrina, PR, Brazil |
| Arlety M. C. | Casale |  | MD | Hospital Universitário da Universidade Federal de São Carlos | São Carlos, SP, Brazil |
| Erika C. N. | Giuliano |  | - | Hospital Universitário da Universidade Federal de São Carlos | São Carlos, SP, Brazil |
| Amarildo B. S. | Oliveira |  | MD | Hospital Universitário de Brasília | Brasília, DF, Brazil |
| Antonio L. | Sarmento | Filho | MD | Hospital Universitário de Brasília | Brasília, DF, Brazil |
| Einstein F. | de Camargos |  | MD, MSc, PhD | Hospital Universitário de Brasília | Brasília, DF, Brazil |
| Larissa F. L. | e Abreu |  | MD | Hospital Universitário de Brasília | Brasília, DF, Brazil |
| Luciana L. L. | Martini |  | MD, MSc, PhD | Hospital Universitário de Brasília | Brasília, DF, Brazil |
| Marco P. D. | Freitas |  | MD, PhD, MSc | Hospital Universitário de Brasília | Brasília, DF, Brazil |
| Vanessa S. | Canossa |  | MD | Hospital Universitário de Brasília | Brasília, DF, Brazil |
| Yan B. | Jardim |  | - | Hospital Universitário de Brasília | Brasília, DF, Brazil |
| Karoline Rodrigues da Silva | Martins |  | MD, MSc | Hospital Universitário Getúlio Vargas | Manaus, AM, Brazil |
| Karlo E. | Moreira |  | - | Hospital Universitário João de Barros Barreto | Belém, PA, Brazil |
| Marina M. G. | Borges |  | MD, MSc | Hospital Universitário João de Barros Barreto | Belém, PA, Brazil |
| Eliana | Pineda |  | MD, MSc | Hospital Universitario Mayor Méderi | Bogotá, Colombia |
| Elly | Morros |  | MD | Hospital Universitario Mayor Méderi | Bogotá, Colombia |
| Luis Carlos | Venegas-Sanabria |  | MD, PhD | Hospital Universitario Mayor Méderi | Bogotá, Colombia |
| Catarina R. F. | do Nascimento |  | MD | Hospital Universitário Onofre Lopes | Natal, RN, Brazil |
| Luana A. C. | Macedo |  | MD | Hospital Universitário Onofre Lopes | Natal, RN, Brazil |
| Marconi E. | Maia | Junior | MD | Hospital Universitário Onofre Lopes | Natal, RN, Brazil |
| Ana Cristina C. | Speranza |  | MD, MSc | Hospital Universitário Pedro Ernesto | Rio de Janeiro, RJ, Brazil |
| Carla M. | Ribeiro |  | MD | Hospital Universitário Pedro Ernesto | Rio de Janeiro, RJ, Brazil |
| Marilia G. S. | Torre |  | MD | Hospital Universitário Pedro Ernesto | Rio de Janeiro, RJ, Brazil |
| Nathalia | Gomes |  | MD | Hospital Universitário Pedro Ernesto | Rio de Janeiro, RJ, Brazil |
| Christiane M. | Santana |  | MD | Hospital Universitário Professor Edgard Santos | Salvador, BA, Brazil |
| Fabia S. O. | Junqueira |  | - | Hospital Universitário Professor Edgard Santos | Salvador, BA, Brazil |
| Jonas Gordilho | Souza |  | MD, PhD | Hospital Universitário Professor Edgard Santos | Salvador, BA, Brazil |
| Manuela O. C. | Magalhães |  | MD, PhD | Hospital Universitário Professor Edgard Santos | Salvador, BA, Brazil |
| Murilo S. S. | Passos |  | - | Hospital Universitário Professor Edgard Santos | Salvador, BA, Brazil |
| Ronald C. | Gomez |  | MD, MSc | Hospital Universitario San Ignacio | Bogotá, Colombia |
| Samir A. | Aruachan |  | MD | Hospital Universitario San Ignacio | Bogotá, Colombia |
| Mirella R. | Bezerra |  | MD, PhD, MSc | Instituto de Medicina Integral Prof Fernando Figueira | Recife, PE, Brazil |
| Ana L. | Kanaji |  | MD, PhD | Instituto do Câncer do Estado de São Paulo | São Paulo, SP, Brazil |
| Theodora | Karnakis |  | MD, PhD | Instituto do Câncer do Estado de São Paulo | São Paulo, SP, Brazil |
| Claudia K. | Suemoto |  | MD, PhD, MSc | Laboratório de Investigação Médica em Envelhecimento (LIM-66), Serviço de Geriatria, Hospital das Clínicas da Faculdade de Medicina, Universidade de São Paulo | São Paulo, SP, Brazil |
| Eduardo | Ferriolli |  | MD, PhD | Laboratório de Investigação Médica em Envelhecimento (LIM-66), Serviço de Geriatria, Hospital das Clínicas da Faculdade de Medicina, Universidade de São Paulo | São Paulo, SP, Brazil |
| Maria Fernanda B. | Roma |  | MD | Laboratório de Investigação Médica em Envelhecimento (LIM-66), Serviço de Geriatria, Hospital das Clínicas da Faculdade de Medicina, Universidade de São Paulo | São Paulo, SP, Brazil |
| Andreyna J. | Rodrigues |  | RN, MSN | Real Hospital Português de Beneficência | Recife, PE, Brazil |
| Camila M. F. D. | Ferreira |  | MD | Real Hospital Português de Beneficência | Recife, PE, Brazil |
| Iolanda G. R. | de Oliveira |  | MD | Real Hospital Português de Beneficência | Recife, PE, Brazil |
| Ivo B. S. | Silva |  | MD | Real Hospital Português de Beneficência | Recife, PE, Brazil |
| Lucas G. | de Andrade |  | MD | Real Hospital Português de Beneficência | Recife, PE, Brazil |
| Luciulo | Melo |  | MD, MSc | Real Hospital Português de Beneficência | Recife, PE, Brazil |
| Milena M. | dos Santos |  | RN | Real Hospital Português de Beneficência | Recife, PE, Brazil |
| Vanessa F. R. | Saraiva |  | MD | Real Hospital Português de Beneficência | Recife, PE, Brazil |
| Walter A. | de Araujo | Junior | MD | Real Hospital Português de Beneficência | Recife, PE, Brazil |
| Flavio F. | Arbex |  | MD, PhD | Santa Casa de Araraquara | Araraquara, SP, Brazil |
| Maria Carolyna F. B. | Arbex |  | MD, MSc | Santa Casa de Araraquara | Araraquara, SP, Brazil |
| Maria J. C. | Souza |  | MD, MS | Santa Casa de Araraquara | Araraquara, SP, Brazil |
